# Comparative Efficacy, Safety, and Costs of Sorafenib vs. Sunitinib as First-Line Therapy for Metastatic Renal Cell Carcinoma: A Systematic Review and Meta-Analysis

**DOI:** 10.3389/fonc.2019.00479

**Published:** 2019-06-21

**Authors:** Huan Deng, Wenfeng Liu, Ting He, Zhengdong Hong, Fengming Yi, Yiping Wei, Wenxiong Zhang

**Affiliations:** ^1^Department of Thoracic Surgery, The First Affiliated Hospital of Nanchang University, Nanchang, China; ^2^Jiangxi Medical College, Nanchang University, Nanchang, China; ^3^Department of Urology, The Second Affiliated Hospital of Nanchang University, Nanchang, China; ^4^Department of Oncology, The Second Affiliated Hospital of Nanchang University, Nanchang, China

**Keywords:** sorafenib, sunitinib, targeted therapy, renal cell carcinoma, meta-analysis

## Abstract

**Purpose:** Sorafenib and sunitinib are extensively used as first-line medications for metastatic renal cell carcinoma (mRCC). This meta-analysis was conducted to assess the antitumor efficacy, toxicity, and costs of the two drugs among mRCC patients.

**Materials and methods:** PubMed, ScienceDirect, Scopus, Web of Science, Ovid MEDLINE, the Cochrane Library, Embase, and Google Scholar were searched for eligible articles. The endpoints consisted of progression-free survival (PFS), overall survival (OS), objective response rate (ORR), adverse effects (AEs), and per-patient-per-month (PPPM) costs.

**Results:** We included 14 studies with 2,925 patients. Both drugs were valid for treating mRCC with equivalent PFS [hazard ratio (HR) = 0.98, 95% confidence interval (CI): 0.88–1.10, *P* = 0.74] and disease control rates [DCRs; risk ratio (RR) = 1.03, 95% CI: 0.98–1.08, *P* = 0.28], but sunitinib had a better OS (HR = 1.10, 95% CI: 1.01–1.20, *P* = 0.04) and higher ORR (HR = 0.66, 95% CI: 0.45–0.97, *P* = 0.03) than sorafenib. Furthermore, sunitinib induced more incidences of severe hematologic AEs (anemia, neutropenia, and thrombocytopenia) and stomatitis/mucositis than sorafenib. In the subanalysis, Asian patients treated with sorafenib reported a longer PFS than those treated with sunitinib (HR = 0.87, 95% CI: 0.83–0.90, *P* = 0.01), and European patients treated with sunitinib had a longer OS than those treated with sorafenib (HR = 1.17, 95% CI: 1.01–1.30, *P* = 0.04). Moreover, the pooled results of the high-quality studies reported a higher ORR with sunitinib than with sorafenib, and medium-quality studies showed a longer OS with sunitinib than with sorafenib.

**Conclusions:** Sunitinib has more benefits (longer OS and better ORR) than sorafenib as a first-line therapy for mRCC. However, sunitinib has higher toxicity than sorafenib. Sorafenib might be more suitable than sunitinib among Asian patients, and sunitinib might be superior to sorafenib in European patients. Nevertheless, more large-scale, high-quality studies are required.

## Introduction

Renal cell carcinoma (RCC) is a common tumor in the urological system, with an expected 73,820 cases and 14,770 deaths in 2019 (1). Moreover, more than 30% of the patients had metastases when initially diagnosed, and 20–40% developed systemic spread after undergoing surgery (2). The National Comprehensive Cancer Network (NCCN) guidelines recommend sunitinib and sorafenib as first-line drugs for metastatic clear cell RCC (mRCC) (3).

Sorafenib and sunitinib are both small-molecule tyrosine kinase inhibitors (TKIs). Both of these drugs have many targets and can inhibit vascular endothelial growth factor (VEGF) and platelet-derived growth factor (PDGF) receptor tyrosine kinases. Furthermore, as a kind of RAF kinase inhibitor, sorafenib can interrupt the RAS/RAF/MEK intracellular signaling pathway (4, 5). A phase II clinical trial has indicated that sorafenib has a better progression-free survival (PFS) than anticipated as therapy for mRCC (6). Sunitinib is also used as a first-line TKI as targeted treatment for mRCC and has shown excellent antitumor efficacy and safety for treating mRCC in a randomized controlled trial (RCT) (NCT00130897) (7). Although both TKIs have shown great benefits for treating mRCC, the best patient profile for the use of the two targeted drugs is still unclear. In a retrospective study, Cai et al. suggested that sorafenib had an equivalent treatment efficacy to but a lower toxicity than sunitinib as first-line therapy for mRCC (8). However, Di Fiore et al. reported that sunitinib had worse survival and more severe clinical adverse events (AEs) for advanced RCC (aRCC) than sorafenib (9).

To address this discrepancy, a meta-analysis of the relevant articles was performed to compare the treatment efficacy, toxicity, and costs of sorafenib and sunitinib and provide evidence-based suggestions for the optimal first-line treatment in patients with mRCC.

## Materials and Methods

This meta-analysis was performed in accordance with the Preferred Reporting Items for Systematic Review and Meta-Analysis (PRISMA) guidelines (Table S1).

### Search Strategies

PubMed, ScienceDirect, Embase, Web of Science, Ovid MEDLINE, the Cochrane Library, Scopus, and Google Scholar were searched up to November 2018 to select relevant studies that compared sorafenib and sunitinib as first-line therapy for mRCC. The following terms were used: “sorafenib,” “sunitinib,” and “renal cell carcinoma.” We used the complete search function in PubMed for the following terms: (sorafenib [MeSH Terms] OR sorafenib [Text Word] OR BAY 545-9085 [Text Word] OR BAY 43-9006 [Text Word] OR Nexavar [Text Word]) AND (sunitinib [MeSH Terms] OR sunitinib [Text Word] OR Sutent [Text Word] OR SU011248 [Text Word]) AND (renal cell carcinoma [MeSH Terms] OR renal cell carcinoma [Text Word]). We also searched the references of all included studies for further eligible articles. All included articles were written in English.

### Inclusion Criteria

We included studies that satisfied the following criteria according to PICOS (population, interventions, comparators, outcomes, and study designs): (1) population: enrolled patients who were diagnosed with mRCC (defined as having distant metastasis); (2) interventions and comparators: compared sorafenib and sunitinib as first-line therapy; (3) outcomes: PFS, overall survival (OS), objective response rate (ORR), disease control rate (DCR), AEs, and per-patient-per-month (PPPM) costs; (4) study designs: RCTs or retrospective observational studies; and (5) were written in English. We excluded reviews without raw data and conference abstracts, case reports, meta-analyses, and articles with repeated data.

### Data Extraction

Two investigators (HD and WZ) extracted the following information independently: first author, the time of publication, country, number of patients in the sorafenib and sunitinib groups, study design, patient characteristics (age, sex, pathological type, pretreatment, and initial dosage), antitumor effectiveness indicators (PFS, OS, ORR, and DCR), number of AEs (all-grade AEs and grade 3–4 AEs), and PPPM costs. The data about the healthcare costs of sorafenib and sunitinib were converted into PPPM costs (US dollar) through mathematical operations. A third researcher (ZH) settled disagreements under various circumstances. We used hazard ratios (HRs), which consider the number and time of events, instead of odds ratios to analyze the PFS and OS. HRs with 95% confidence intervals (CIs) were obtained directly if a Cox multivariate survival analysis was performed. Otherwise, HRs and 95% CIs were extracted from the Kaplan–Meier curves in accordance with the protocol from Tierney et al. (10).

### Quality Assessment

We evaluated the quality of the retrospective observational studies through the 9-point Newcastle–Ottawa Scale, which included a questionnaire on the following three major aspects: selection, comparability, and exposure. A total score of 8–9 points suggested that a study was high quality, and a study with 6–7 points was medium quality (11).

### Statistical Analysis

We performed this meta-analysis using Review Manager (version 5.2) and STATA (version 12.0). HRs and 95% CIs were used to analyze the PFS and OS (HR < 1 supports the sorafenib group; HR > 1 supports the sunitinib group). Risk ratios (RRs) and 95% CIs were used to analyze the ORR, DCR (RR < 1 supports the sunitinib group; RR > 1 supports the sorafenib group), and AEs (RR < 1 supports the sorafenib group; RR > 1 supports the sunitinib group). Weighted mean differences (WMDs) with 95% CIs were used to analyze the PPPM. Subgroup analyses of the PFS, OS, and ORR were performed to determine if these outcomes vary based on nationality, initial dosage, and study quality. Heterogeneity was assessed through the χ^2^ test and *I*^2^ statistic. If *I*^2^ > 50% or *P* < 0.1, then the study showed significant heterogeneity. We would use the random-effects model rather than the fixed-effects model in all results (including subanalysis) in case we draw misleading conclusions. To enhance robustness, sensitivity analyses of the PFS, OS, ORR, and DCR were performed to determine if these effects were variables. We assessed publication bias through Begg's test and Egger's test. Significant differences were considered as *P* < 0.05.

## Results

### Search Results and Quality Evaluation

**Figure 1:**
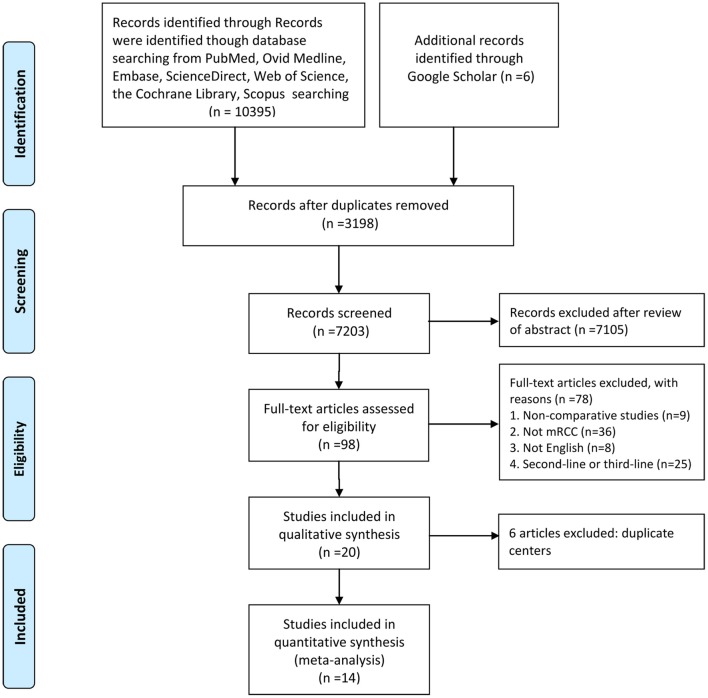
Flowchart of the study selection process.

Figure 1 shows the study selection process. An eventual 14 studies involving 2,925 patients (sorafenib, 1,150; sunitinib, 1,775) were selected for this meta-analysis (9, 12–24). All included articles were retrospective observational studies. In fact, two studies originated from the same patient population; one study reported the PPPM, and the other reported anti-efficacy and toxicity (22, 23). Nine articles were considered high quality (two articles scored 9 points, seven articles scored 8 points). Five articles were considered medium quality (three articles scored 7 points, two articles scored 6 points; Table S2). Table 1 lists the baseline characteristics and key evaluation indicators of all included articles.

**Table 1:** Characteristics of 14 included studies.

**References**	**Country**	**Study period**	**Pretreatment**	**Groups**	**Patients (*n*)**	**Initial dosage**		**Median age (y)**	**CCRCC (%)**	**Design**	**Score**
						**So**	**Su**				
Sheng et al. (12)	China	2006.11–2015.03	CN	So vs. Su	169/166	400 mg BID in a 4-week cycle	50 mg daily in a 6-week cycle (4/2)	54.0/55.0	81/85	RS	8
Zhang et al. (13)	China	2006.09–2014.12	NPT	So vs. Su	483/362	400 mg BID in a 4-week cycle	50 mg daily in a 6-week cycle (4/2)	NA	85/91	RS	9
Cai et al. (8)	China	2006.03–2015.07	NPT, CT	So vs. Su	110/74	400 mg BID in a 4-week cycle	50 mg daily in a 6-week cycle (4/2)	NA	96/96	RS	8
La Vine et al. (14)	USA	2005.12–2008.03	NPT	So vs. Su	24.0/10	400 mg BID in a 4-week cycle	50 mg daily in a 6-week cycle (4/2)	NA	NA	RS	7
Harrison et al. (15)	USA	2007.01–2011.01	NPT	So vs. Su	42/188	NA	NA	63.6/63.7 a	62/66	RS	8
Derbel Miled et al. (16)	France	2005.03–2009.10	NPT	So vs. Su	24/24	NA	NA	75.7/75.3	88/83	RS	8
Maroun et al. (17)	France	2012.11–2014.09	NA	So vs. Su	19/238	NA	NA	59.0/65.0 a	NA	RS	6
Levy et al. (18)	France	2005.01–2009.12	NA	So vs. Su	60/127	NA	NA	60.0/58.0	92/80	RS	6
Busch et al. (19)	German	NA	RN	So vs. Su	7/28	400 mg BID in a 4-week cycle	37.5 mg in a 6-week cycle (4/2)	NA	NA	RS	7
Park et al. (20)	Korea	2005.04–2011.03	NPT, IM, CH, ME	So vs. Su	49/220	400 mg BID in a 4-week cycle	50 mg daily in a 6-week cycle (4/2)	62.0/56.5	88/80	RS	8
Ishihara et al. (21)	Japan	2007.01–2016.06	NPT, CT	So vs. Su	43/91	200 mg BID in a 4-week cycle	50 mg/day in a 4/2 and 2/1 schedule	65.0/68.0	72/75	RS	9
Choueiri et al. (22)	USA, Israel	2003.04–2008.06	CT, RT, CH	So vs. Su	62/57	94% patients 400 mg BID in a 4-week cycle	84% patients 50 mg/day in a 4/2 schedule	60.0/58.0	NA	RS	8
Choueiri et al. (23)	USA, Israel	2003.04–2008.06	CT, CH, IM	So vs. Su	62/57	94% patients 400 mg BID in a 4-week cycle	84% patients 50 mg/day in a 4/2 schedule	60.0/58.0	NA	RS	8
Santoni et al. (24)	Italy	2005.01–2013.07	RN, CH, IM	So vs. Su	58/190	400 mg BID in a 4-week cycle	50 mg daily in a 6-week cycle (4/2)	NA	100/100	RS	7

*CCRCC, clear cell RCC; NPT, nephrectomy; RN, radical nephrectomy; IM, immunotherapy; CT, cytokine therapy; RT, radiation therapy; CH, chemotherapy; ME, metastasectomy; So, sorafenib; Su, sunitinib; 4/2, dosing for 4 weeks followed by 2 weeks off; RS, retrospective study; 2/1, dosing for 2 weeks followed by 1 week off; RS, retrospective study; NA, not available; a, mean*.

### Antitumor Efficacy

The antitumor effectiveness regarding PFS, OS, ORR, and DCR between the two groups was evaluated.

**Figure 2:**
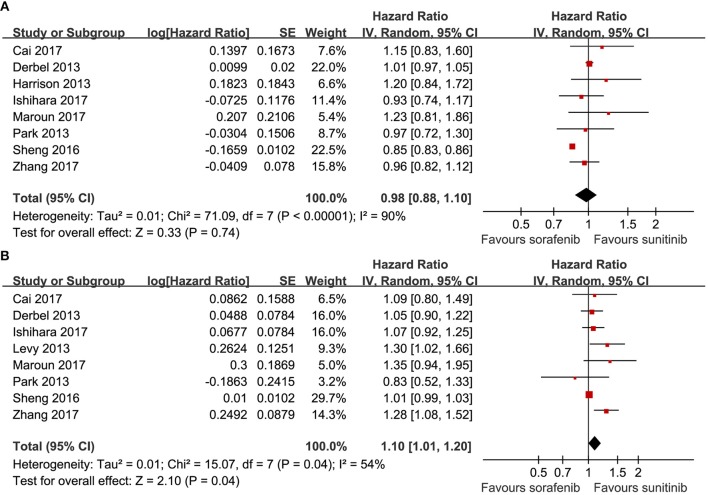
Forest plots of the PFS **(A)** and OS **(B)** associated with sorafenib vs. sunitinib.

Eight articles compared the PFS (heterogeneity: *P* < 0.00001, *I*^2^ = 90%). Significant differences were not found between sorafenib and sunitinib (HR = 0.98, 95% CI: 0.88–1.10, *P* = 0.74; Figure 2A).

Eight articles compared the OS (heterogeneity: *P* = 0.04, *I*^2^ = 54%). Sunitinib-treated patients had a better OS than sorafenib-treated patients (HR = 1.10, 95% CI: 1.01–1.20, *P* = 0.04; Figure 2B).

**Figure 3:**
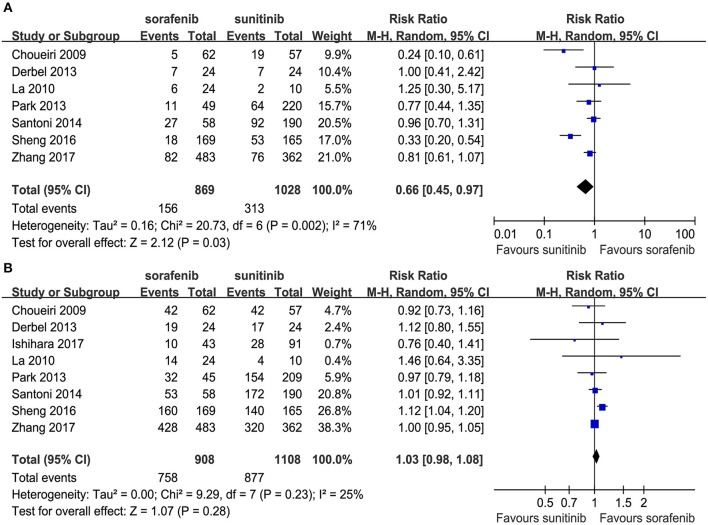
Forest plots of the ORR **(A)** and DCR **(B)** associated with sorafenib vs. sunitinib.

Seven articles compared the ORR (heterogeneity: *P* = 0.002, *I*^2^ = 71%). The sunitinib group had a higher ORR than the sorafenib group (RR = 0.66, 95% CI: 0.45–0.97, *P* = 0.03; Figure 3A).

Eight articles compared the DCR (heterogeneity: *P* = 0.23, *I*^2^ = 25%). Significant differences were not found between the groups (RR = 1.03, 95% CI: 0.98–1.08, *P* = 0.28; Figure 3B).

### Toxicity

We compared the toxicity between sorafenib and sunitinib based on the total number of AEs and performed subgroup analyses of the 10 most common toxic events.

**Figure 4:**
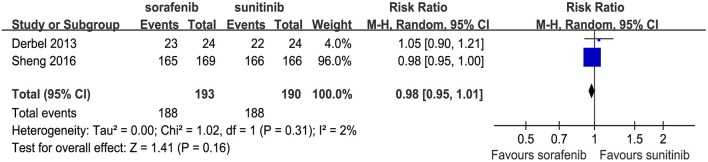
Forest plots of the RR of total AEs associated with sorafenib vs. sunitinib.

Two articles compared the total number of AEs (heterogeneity: *P* = 0.31, *I*^2^ = 2%). No significant differences were found between the two TKIs (RR = 0.98, 95% CI: 0.95–1.01, *P* = 0.16; Figure 4).

**Figure 5:**
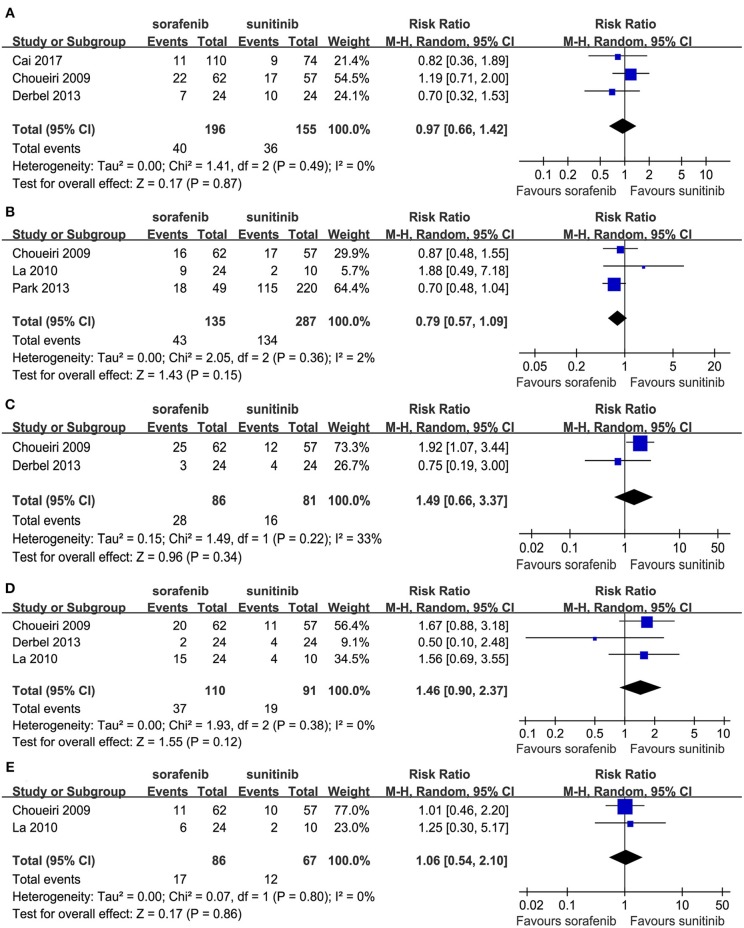
Forest plots of the RR of drug reductions **(A)**, drug reductions due to serious AEs **(B)**, drug interruption **(C)**, drug interruption due to serious AEs **(D)**, and drug discontinuations due to serious AEs **(E)** associated with sorafenib vs. sunitinib.

Some patients experienced reductions, interruptions, or discontinuations with their drug treatments. Three studies compared drug reductions; no significant difference was found between the two TKIs (RR = 0.97, 95% CI: 0.66–1.42, *P* = 0.87; Figure 5A). Three studies compared the drug reductions due to serious AEs; no significant difference was found between the two groups (RR = 0.79, 95% CI: 0.57–1.09, *P* = 0.15; Figure 5B). Two studies compared drug interruptions; no significant difference was found between the two groups (RR = 1.49, 95% CI: 0.66–3.37, *P* = 0.34; Figure 5C). Three studies compared drug interruptions due to serious AEs; no significant difference was found between the two groups (RR = 1.46, 95% CI: 0.90–2.37, *P* = 0.12; Figure 5D). Two studies compared drug discontinuations due to serious AEs; no significant difference was found between the two groups (RR = 1.06, 95% CI: 0.54–2.10, *P* = 0.86; Figure 5E).

In the subgroup analyses of the 10 most common AEs (in order of incidence: hand–foot syndrome, diarrhea, nausea/vomiting, fatigue/asthenia, neutropenia, rash, thrombocytopenia, hypertension, anemia, and stomatitis/mucositis), the outcomes of these all-grade AEs indicated that there were no significant differences in the incidence of hand–foot syndrome, diarrhea nausea/vomiting, rash, hypertension, and anemia between sorafenib and sunitinib. For all-grade AEs, sunitinib had higher incidences of fatigue/asthenia (RR = 0.75, 95% CI: 0.63–0.89, *P* = 0.001), neutropenia (RR = 0.33, 95% CI: 0.23–0.48, *P* < 0.00001), thrombocytopenia (RR = 0.27, 95% CI: 0.20–0.37, *P* < 0.00001), and stomatitis/mucositis (RR = 0.14, 95% CI: 0.03–0.60, *P* = 0.008, Table 2) than sorafenib. The outcomes of grade 3–4 AEs demonstrated that there were no significant differences for hand–foot syndrome, diarrhea, nausea/vomiting, fatigue/asthenia, rash, and hypertension between the two groups. For grade 3–4 AEs, sunitinib had more neutropenia (RR = 0.20, 95% CI: 0.05–0.73, *P* = 0.01), anemia (RR = 0.37, 95% CI: 0.08–0.88, *P* = 0.03), thrombocytopenia (RR = 0.11, 95% CI: 0.04–0.26, *P* < 0.00001), and stomatitis/mucositis (RR = 0.62, 95% CI: 0.40–0.98, *P* = 0.04, Table 3) than sorafenib.

**Table 2:** Top 10 adverse effects (all grade) associated with sorafenib vs. sunitinib.

**Adverse effects**	**The number of study**	**Sorafenib group (event/total)**	**Sunitinib group (event/total)**	**RR (95% CI)**	***P* value**	**Heterogeneity**	
						***I*^**2**^ (%)**	***P* value**
Hand–foot syndrome	3	195/341	157/297	1.07 [0.94, 1.23]	0.32	0	0.71
Diarrhea	3	190/341	140/297	1.30 [0.85, 1.97]	0.23	82	0.004
Nausea/Vomiting	3	140/341	147/297	0.84 [0.71, 1.00]	0.05	36	0.21
Fatigue/Asthenia	3	129/341	151/297	0.75 [0.63, 0.89]	0.001	0	0.61
Neutropenia	1	30/169	88/166	0.33 [0.23, 0.48]	<0.00001	NA	NA
Rash	2	91/231	60/223	1.69 [0.86, 3.31]	0.13	72	0.06
Thrombocytopenia	2	39/279	129/240	0.27 [0.20, 0.37]	<0.00001	0	0.99
Hypertension	3	90/341	95/297	0.84 [0.67, 1.07]	0.16	43	0.18
Anemia	2	34/279	67/240	0.59 [0.10, 3.57]	0.56	94	<0.0001
Stomatitis/Mucositis	1	2/62	13/57	0.14 [0.03, 0.60]	0.008	NA	NA

*NA, not available*.

**Table 3:** Top 10 adverse effects (grade 3–4) associated with sorafenib vs. sunitinib.

**Adverse effects**	**The number of study**	**Sorafenib group (event/total)**	**Sunitinib group (event/total)**	**RR (95% CI)**	***P* value**	**Heterogeneity**	
						***I*^**2**^ (%)**	***P* value**
Hand–foot syndrome	4	46/390	73/517	1.13 [0.57, 2.27]	0.73	54	0.09
Diarrhea	5	17/414	24/527	0.81 [0.44, 1.50]	0.50	0	0.87
Nausea/Vomiting	5	3/414	8/527	0.38 [0.07, 2.12]	0.27	39	0.18
Fatigue/Asthenia	5	31/414	42/541	0.81 [0.56, 1.19]	0.29	0	0.75
Neutropenia	3	9/242	80/410	0.20 [0.05, 0.73]	0.01	45	0.16
Rash	4	21/304	14/453	1.58 [0.39, 6.45]	0.53	62	0.05
Thrombocytopenia	4	4/352	89/484	0.11 [0.04, 0.26]	<0.00001	0	0.75
Hypertension	5	18/414	51/541	0.57 [0.32, 1.00]	0.05	26	0.25
Anemia	3	7/328	43/460	0.37 [0.08, 0.88]	0.03	41	0.19
Stomatitis/Mucositis	5	52/473	97/633	0.62 [0.40, 0.98]	0.04	13	0.33

### PPPM Costs

We assessed the costs between the two groups based on the PPPM. Only one included article provided mean and standard deviation values and reported that sorafenib had lower PPPM costs than sunitinib ($6,990.36 ± 3,073.11 vs. $7,944.91 ± 2,993.36) (23).

### Subgroup Analysis

To determine if the treatment efficacy of sorafenib vs. sunitinib changed over time, the pooled results of PFS, OS, and ORR were calculated according to nationality, initial dosage, and study quality (Table 4). Interestingly, Asian patients treated with sorafenib had a longer PFS than European patients (HR = 0.87, 95% CI: 0.83–0.90, *P* = 0.01); European studies (all from France) indicated that sunitinib led to a longer OS than sorafenib (HR = 1.17, 95% CI: 1.01–1.30, *P* = 0.04). The pooled results of the high-quality studies indicated that sunitinib had a higher ORR than sorafenib (HR = 0.57, 95% CI: 0.35–0.93, *P* = 0.02), and the medium-quality studies showed that sunitinib led to a longer OS than sorafenib (HR = 1.32, 95% CI: 1.07–1.61, *P* = 0.008).

**Table 4:** Subgroup analysis for PFS, OS, and ORR.

**Group**	**PFS**				**OS**				**ORR**			
	**No. of studies**	**HR (95% CI)**	***P***	***I*^**2**^ (%)**	**No.of studies**	**HR (95% CI)**	***P***	***I*^**2**^ (%)**	**No. of studies**	**RR (95% CI)**	***P***	***I*^**2**^ (%)**
**Total**	8	0.98 [0.88, 1.10]	0.74	90	8	1.10 [1.01, 1.20]	0.04	54	7	0.66 [0.45, 0.97]	0.03	71
**NATION**												
Asia	5	0.87 [0.83, 0.90]	0.01	44	5	1.07 [0.96, 1.19]	0.20	54	3	0.60 [0.34, 1.05]	0.07	80
Europe	2	1.01 [0.97, 1.05]	0.56	0	3	1.17 [1.01, 1.30]	0.04	34	2	0.97 [0.72, 1.30]	0.82	0
North America	1	1.20 [0.84, 1.72]	0.32	NA	NA	NA	NA	NA	2	0.50 [0.10, 2.50]	0.40	73
**INITIAL DOSAGE**												
Standard dosage	4	0.92 [0.82, 1.04]	0.18	54	4	1.08 [0.92, 1.26]	0.35	63	5	0.71 [0.48, 1.05]	0.09	72
NA	3	1.01 [0.98, 1.05]	0.49	0	3	1.23 [1.04, 1.46]	0.02	0	1	1.00 [0.41, 2.42]	1.00	NA
Su group uses 4/2 and 2/1 schedule	1	0.93 [0.74, 1.17]	0.54	NA	1	1.07 [0.92, 1.25]	0.39	NA	NA	NA	NA	NA
Most patients use standard dosage	NA	NA	NA	NA	NA	NA	NA	NA	1	0.24 [0.10, 0.61]	0.002	NA
**STUDY QUALITY^a^**												
High quality	7	0.97 [0.87, 1.08]	0.58	91	6	1.07 [0.97, 1.17]	0.17	43	5	0.57 [0.35, 0.93]	0.02	74
Medium quality	1	1.23 [0.81, 1.86]	0.33	NA	2	1.32 [1.07, 1.61]	0.008	0	2	0.97 [0.72, 1.32]	0.86	0

Su, sunitinib; 4/2, 4 weeks on and 2 weeks off; 2/1, 2 weeks on and 1 week off; standard dosage, sunitinib group uses 4/2 schedule and sorafenib group uses a 4-week cycle; NA, not available.

a *study quality was evaluated according to Newcastle–Ottawa Scale. Total scores of 8–9 points is high quality and 6–7 points is medium quality*.

### Sensitivity Analysis

The PFS (Figure S1A) and OS (Figure S1B) were all robust, with consistent findings from the sensitivity analysis. In addition, the ORR (Figure S2A) and DCR (Figure S2B) were all robust, and the sensitivity analysis showed that no estimates were beyond the 95% CIs.

### Publication Bias

No proof of publication bias was found for the PFS (Begg's test, *P* = 0.386, Egger's test, *P* = 0.187; Figure S3A), OS (Begg's test, *P* = 0.803; Egger's test, *P* = 0.071; Figure S3B), ORR (Begg's test, *P* = 1.000; Egger's test, *P* = 0.651; Figure S4A), and DCR (Begg's test, *P* = 0.711; Egger's test, *P* = 1.000; Figure S4B).

## Discussion

This may be the first meta-analysis to compare the antitumor efficacy, toxicity, and costs between these two TKIs as first-line treatment for mRCC. Our analysis of 14 medium- to high-quality studies shows that sunitinib was associated with more benefits (improved OS and better ORR) than sorafenib as first-line therapy for mRCC. However, sunitinib was associated with more all-grade and grade 3–4 neutropenia, thrombocytopenia, and stomatitis/mucositis than sorafenib. However, sorafenib might have lower PPPM costs than sunitinib, although only one study reported PPPM costs. In the subgroup analyses, Asian patients using sorafenib had a longer PFS than those using sunitinib, and European patients using sunitinib had a superior OS than those using sorafenib as first-line therapy for mRCC; the pooled outcomes of the high-quality studies reported that sunitinib had a higher ORR than sorafenib, and the medium-quality studies showed that sunitinib had a longer OS than sorafenib.

Antitumor efficacy is the most predominant cornerstone to consider when comparing sorafenib and sunitinib. The pooled analysis indicated that there were no significant differences between the two groups in PFS and DCR. However, sunitinib had a better OS than sorafenib. In a retrospective analysis with 251 consecutive patients, Levy et al. reported that sunitinib-treated patients were associated with a longer median OS than sorafenib-treated patients (26.3 vs. 16.4 months) (18). This finding was also confirmed by the subgroup analysis of medium-quality studies for OS, which showed that sunitinib had a better OS than sorafenib (HR = 1.32, 95% CI: 1.07–1.61, *P* = 0.008). Moreover, sunitinib had a higher ORR than and a similar DCR to sorafenib. In other words, although sorafenib-treated patients had a lower ORR, they had more stable disease (SD), which is also considered as a kind of disease control. SD is defined as when the total length of the baseline tumor lesions is reduced in size but does not reach 30% of the original size or increases <20% in size, based on RECIST (Response Evaluation Criteria in Solid Tumors) version 1.1 (25). Furthermore, a retrospective study of the 5-year experiences of two large oncology centers demonstrated that among patients with mRCC, sorafenib led to more SD than sunitinib (69% vs. 45%) (22). Similarly, a large single-center retrospective study indicated that no significant difference was found in terms of preventing progressive disease (PD), although sunitinib was associated with higher objective responses than sorafenib (12). Furthermore, the subgroup analysis of the ORR in the high-quality studies showed that sunitinib had a better ORR than sorafenib (HR = 0.57, 95% CI: 0.35–0.93, *P* = 0.02). Notably, the subgroup analysis showed that the Asian studies reported a longer PFS (Table 4), which suggests that Asian patients using sorafenib as first-line therapy for mRCC might achieve better antitumor efficacy than those using sorafenib. Additionally, the European studies reported a better OS (Table 4), which demonstrates that sunitinib might provide superior antitumor efficacy for European patients compared to sorafenib. Undoubtedly, these positive findings of subanalysis have the effect of revealing the tendency. Our conclusions need to be accepted carefully, especially the findings of the subanalysis. More high-impact, large-sample studies are required to verify these conclusions.

Drug toxicity is an indispensable factor when choosing between sorafenib and sunitinib. Although the incidence of all-grade AEs were not significantly different between the two drugs (Figure 4), sunitinib was associated with more severe AEs, especially grade 3–4 hematologic AEs, than sorafenib (Table 3). Within the grade 3–4 AEs, higher rates of stomatitis/mucositis, anemia, neutropenia, and thrombocytopenia were observed among patients using sunitinib than those using sorafenib. A probable reason might be the use of an inappropriate dose and schedule alterations when using sunitinib. In fact, a phase II RCT showed that therapy with 50 mg sunitinib daily using a 2/1 dosing schedule (2 weeks on and 1 week off) was associated with less toxicity and superior tolerability among patients with mRCC than a standard 4/2 schedule (4 weeks on and 2 weeks off) (NCT00570882) after 30 months of follow-up (26). Similarly, a retrospective study of 99 patients showed that sunitinib on a 2/1 schedule had fewer grade 3–4 AEs among Chinese patients with mRCC than a 4/2 schedule (27). In a systematic review of the side effects of TKIs, Bhojani et al. found that sunitinib led to the most grade 3/4 AEs, and sorafenib led to the fewest grade 3–4 AEs out of sorafenib, sunitinib, and temsirolimus (28). Furthermore, although the two targeted drugs were equally effective among elderly patients with aRCC, sunitinib was less well-tolerated than sorafenib (16). Notably, this difference in toxicity might be of vital importance for patients, as treatment is administered continuously over months. Although toxicity might not reduce the survival time, it could significantly decrease patient compliance, influence the patients' quality of life, and undermine treatment efficacy. The timely prevention, recognition, and prompt management of AEs are essential to avoid unnecessary dose reductions, interruption, or discontinuation of treatment, which may impair the antitumor efficacy.

The cost effects are also a significant factor when choosing between the two TKIs. The only included study that provided mean and standard deviation values reported that patients treated with sorafenib had lower PPPM costs than sunitinib as first-line treatment for mRCC ($6,990.36 ± 3,073.11 vs. $7,944.91 ± 2,993.36) (23). In a retrospective claims database analysis, Duh et al. found that the mean medical costs of sorafenib were less than that of sunitinib in the treatment of aRCC ($6,998 and $8,213, respectively) (29). Similarly, in an analysis of 18 American community oncology clinics, Chen et al. reported that sunitinib was associated with higher PPPM costs than sorafenib ($9,417.35 ± 670.78 vs. $7,992.48 ± 682.29) (30). Although the costs of the two drugs were not greatly different, sorafenib therapy might help relieve the financial pressure on patients and their families to some extent, extend the time of using TKIs while providing equivalent treatment efficacy as sunitinib, and even relieve the psychological burden of patients facing high medical expenses, especially for patients from low-income families or developing countries.

Several limitations should be addressed when considering our results. First, the lack of RCTs weakens the quality of these outcomes. Second, the significant heterogeneity of some comparisons (PFS and ORR) may impair the reliability of these results. Third, the number of patients in the two groups was not large enough, which might have resulted in relatively unreliable estimates. Fourth, selection bias may exist because the included articles were limited to the literature published in English. Fifth, our subgroup analysis of Asia only included three Asian countries (China, Korea, and Japan), and the Europe analysis only included two European countries (France and Italy), which may decrease the representativeness of the positive subgroup results. Sixth, we could not completely control for confounding factors (pathological status, pretreatment, and metastatic sites) as they were unavailable for some studies, but these factors could influence the final results.

## Conclusions

Our meta-analysis demonstrates that sunitinib has more benefits (longer OS and better ORR) than sorafenib as first-line therapy for mRCC. However, sunitinib has higher toxicity and higher PPPM costs than sorafenib. The subanalysis reveals that sorafenib might be more suitable among Asian patients, and European patients using sunitinib might achieve better survival than those using sorafenib. Nevertheless, the inherent limitations of this meta-analysis mean that additional high-impact studies with large samples are needed to better determine the roles of sorafenib and sunitinib under complicated clinical conditions.

## Data Availability

No datasets were generated or analyzed for this study.

## Author Contributions

HD, ZH, WL, TH, and WZ conceived and designed the study. HD, ZH, and FY performed the literature search, data extraction, quality assessment for the included studies, and statistical analysis. HD and WZ wrote the paper. YW reviewed and edited the manuscript. All authors read and approved the manuscript.

### Conflict of Interest Statement

The authors declare that the research was conducted in the absence of any commercial or financial relationships that could be construed as a potential conflict of interest.

## Supplementary Material

Table S1 PRISMA 2009 checklist.

Table S2 Quality assessment of all included studies.

Figure S1 Sensitivity analysis of PFS **(A)** and OS **(B)** associated with sorafenib vs. sunitinib.

Figure S2 Sensitivity analysis of ORR **(A)** and DCR **(B)** associated with sorafenib vs. sunitinib.

Figure S3 Begg's and Egger's tests for comparisons of OS **(A)** and PFS **(B)** associated with sorafenib vs. sunitinib.

Figure S4 Begg's and Egger's tests for comparisons of ORR **(A)** and DCR **(B)** associated with sorafenib vs. sunitinib.

## Abbreviations

mRCC: metastatic renal cell carcinoma
aRCC: advanced renal cell carcinoma
AEs: adverse effects
DCR: disease control rate
HRs: hazard ratios
RRs: risk ratios
PPPM: per-patient-per-month costs
ORR: objective response rate
OS: overall survival
PFS: progression-free survival
PRISMA: preferred reporting items for systematic review and meta-analysis
RCT: randomized controlled trial
TKI: tyrosine kinase inhibitor
WMD: weighted mean difference
CIs: confidence intervals
NCCN: National Comprehensive Cancer Network
VEGF: vascular endothelial growth factor
PDGF: platelet-derived growth factor
SD: stable disease
PD: progressive disease
PICOS, population, interventions, comparators: outcomes and study designs
RECIST: Response Evaluation Criteria in Solid Tumors.
