# Supplementary material for: Comparative Efficacy, Safety, and Costs of Sorafenib vs. Sunitinib as First-Line Therapy for Metastatic Renal Cell Carcinoma: A Systematic Review and Meta-Analysis
Source: Front Oncol. 2019 Jun 21;9:479. doi: 10.3389/fonc.2019.00479 (PMC6598399; doi:10.3389/fonc.2019.00479)
Supplement: Table S2 — Quality assessment of all included studies. [file Table_2.DOCX]

**Table S2** Quality assessment of all included studies

| **Study** | | **Selection** | **Comparability** | **Exposure** | **Quality (score)** |
| --- | --- | --- | --- | --- | --- |
| 2017 | Cai [8] | ★★★ | ★★ | ★★★ | 8 |
| 2016 | Sheng [12] | ★★★ | ★★ | ★★★ | 8 |
| 2017 | Zhang [13] | ★★★★ | ★★ | ★★★ | 9 |
| 2010 | La [14] | ★★★ | ★★ | ★★ | 7 |
| 2013 | Harrison [15] | ★★★★ | ★★ | ★★ | 8 |
| 2013 | Derbel [16] | ★★★ | ★★ | ★★★ | 8 |
| 2017 | Maroun [17] | ★★★ | ★ | ★★ | 6 |
| 2013 | Levy [18] | ★★★ | ★ | ★★ | 6 |
| 2011 | Busch [19] | ★★★ | ★★ | ★★ | 7 |
| 2013 | Park [20] | ★★★★ | ★★ | ★★ | 8 |
| 2017 | Ishihara [21] | ★★★★ | ★★ | ★★★ | 9 |
| 2009 | Choueiri [22] | ★★★ | ★★ | ★★★ | 8 |
| 2012 | Choueiri [23] | ★★★ | ★★ | ★★★ | 8 |
| 2014 | Santoni [24] | ★★★ | ★★ | ★★ | 7 |
