# Supplementary figures and images for: Comparative Efficacy, Safety, and Costs of Sorafenib vs. Sunitinib as First-Line Therapy for Metastatic Renal Cell Carcinoma: A Systematic Review and Meta-Analysis
Source: Front Oncol. 2019 Jun 21;9:479. doi: 10.3389/fonc.2019.00479 (PMC6598399; doi:10.3389/fonc.2019.00479)

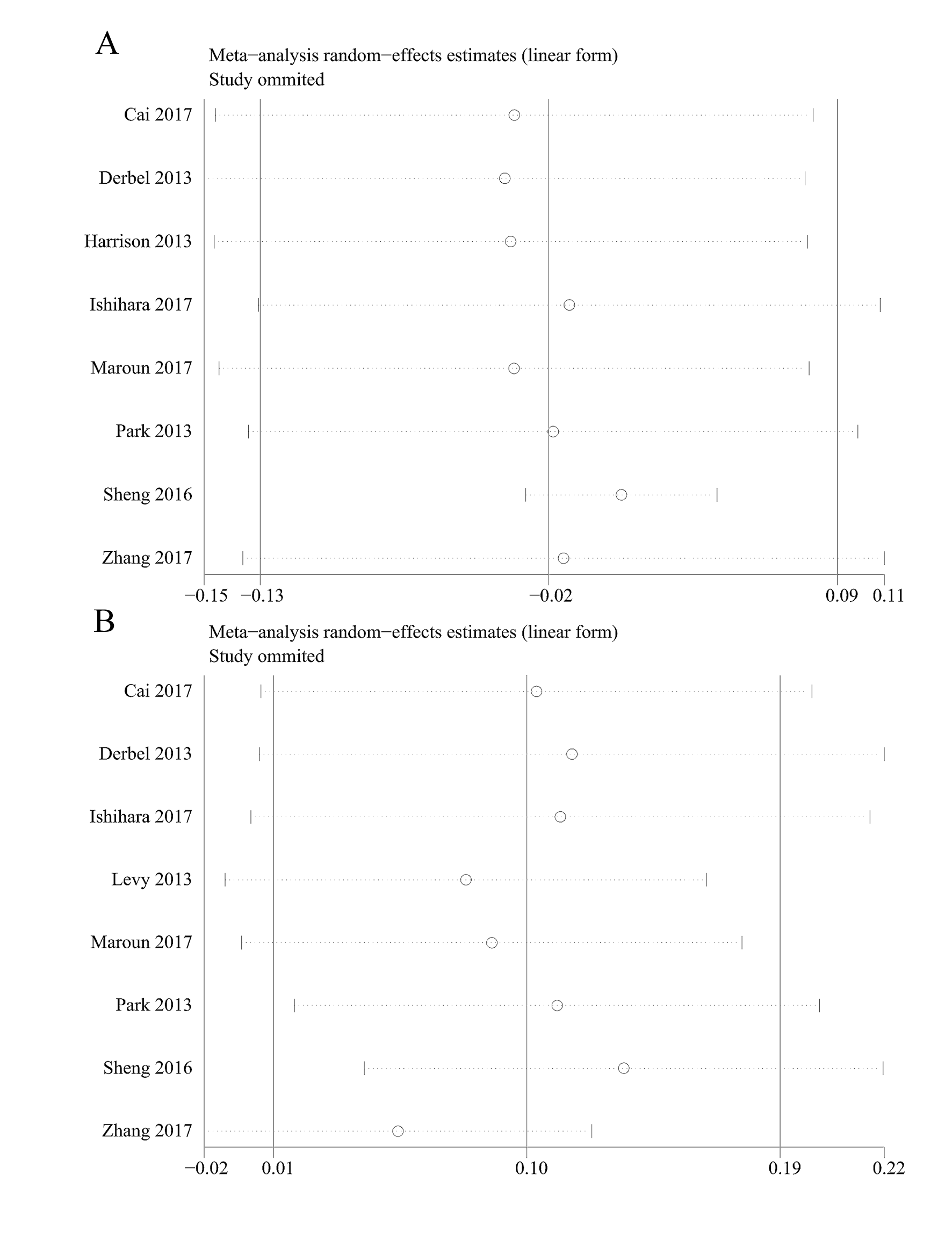

Supplement: Figure S1 — Sensitivity analysis of PFS (A) and OS (B) associated with sorafenib vs. sunitinib. [file Image_1.TIF]

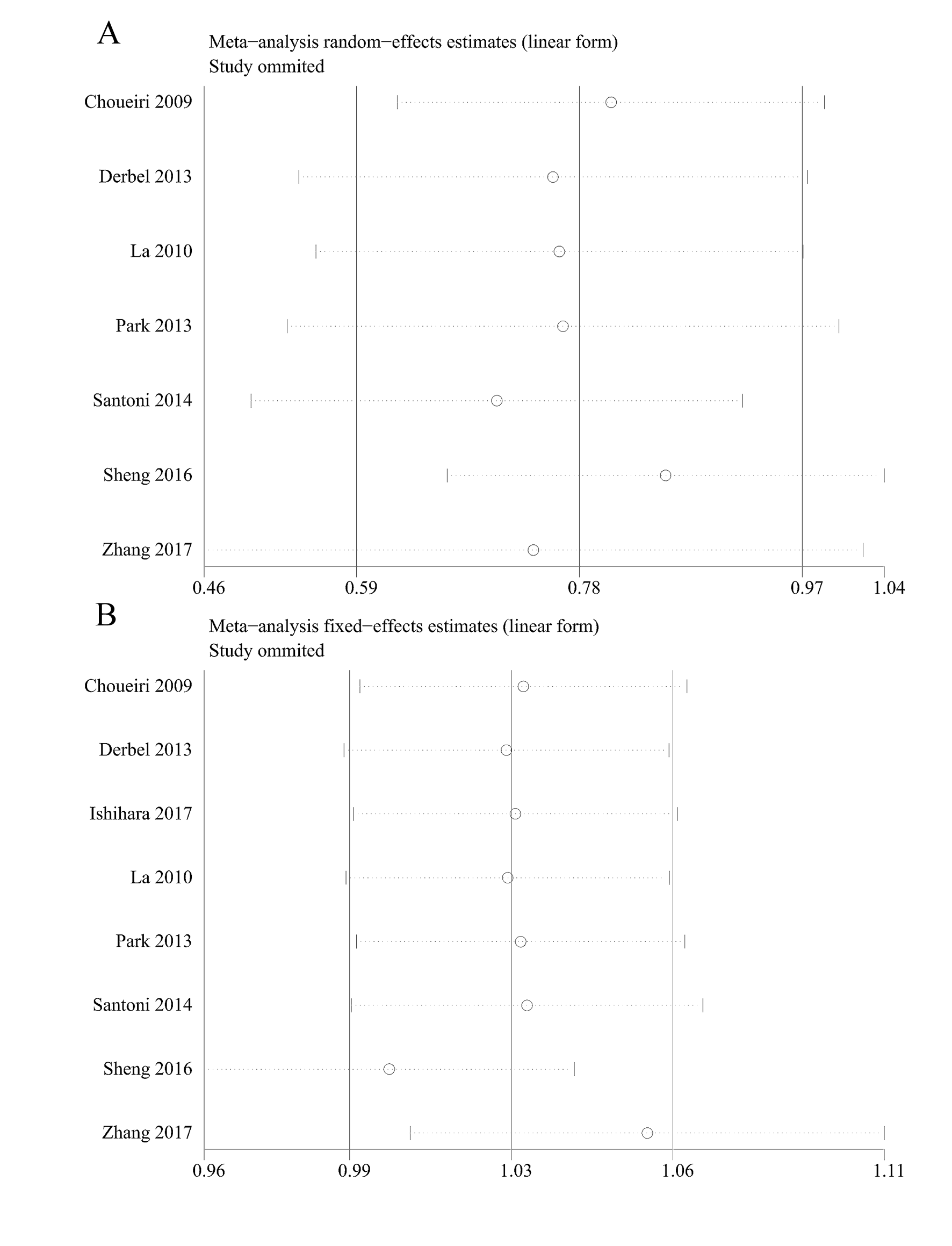

Supplement: Figure S2 — Sensitivity analysis of ORR (A) and DCR (B) associated with sorafenib vs. sunitinib. [file Image_2.TIF]

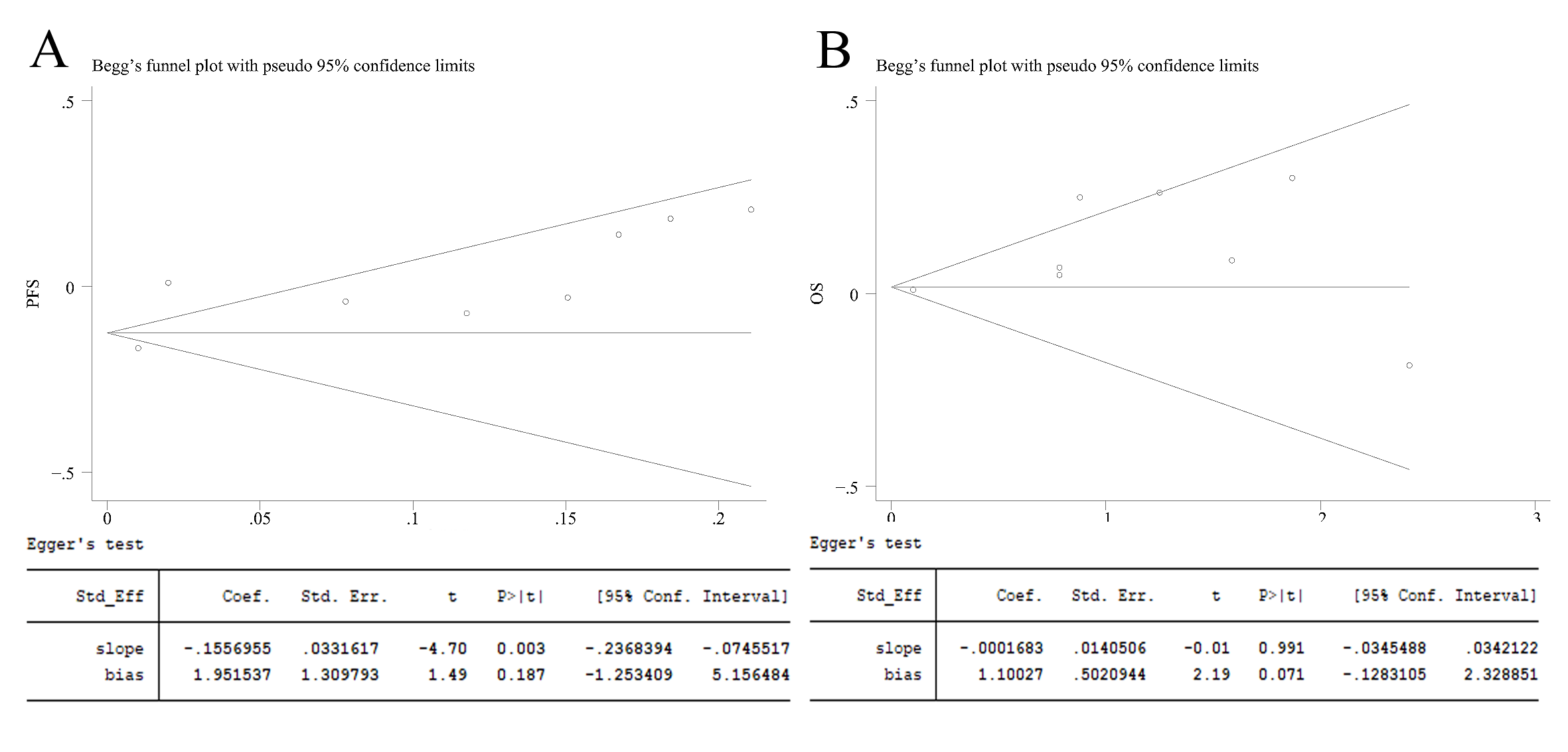

Supplement: Figure S3 — Begg's and Egger's tests for comparisons of OS (A) and PFS (B) associated with sorafenib vs. sunitinib. [file Image_3.tif]

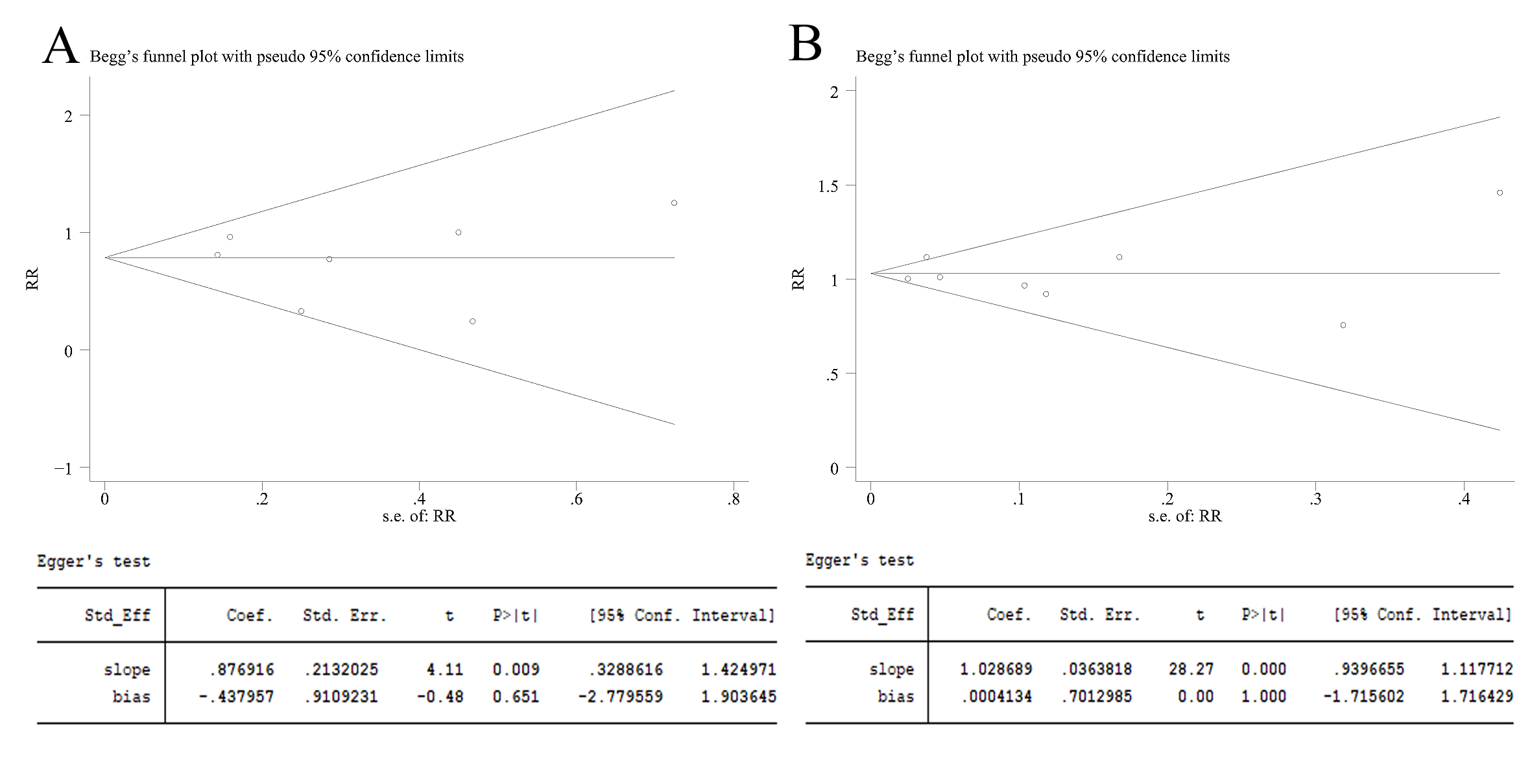

Supplement: Figure S4 — Begg's and Egger's tests for comparisons of ORR (A) and DCR (B) associated with sorafenib vs. sunitinib. [file Image_4.TIF]
